# Cholangiocellular carcinoma occurrence after HCV eradication therapy: case series and review of the literature

**DOI:** 10.25122/jml-2022-0102

**Published:** 2022-10

**Authors:** Razvan Cerban, Adina Croitoru, Gabriel Becheanu, Speranta Iacob, Carmen Ester, Mihaela Ghioca, Mugur Grasu, Radu Dumitru, Carmen Preda, Madalina Florescu, Liliana Gheorghe

**Affiliations:** 1 Faculty of Medicine, Carol Davila University of Medicine and Pharmacy, Bucharest, Romania; 2 Center for Digestive Disease and Liver Transplantation, Fundeni Clinical Institute, Bucharest, Romania; 3 Department of Oncology, Fundeni Clinical Institute, Bucharest, Romania; 4 Pathology Department, Fundeni Clinical Institute, Bucharest, Romania; 5 Radiology Department, Fundeni Clinical Institute, Bucharest, Romania; 6 Department of Gastroenterology, Sfânta Maria Clinical Hospital, Bucharest, Romania

**Keywords:** hepatitis C virus, cholangiocellular carcinoma, sustained virological response

## Abstract

Hepatitis C viral (HCV) treatment has rapidly advanced with the use of direct-acting antivirals (DAA), and many patients achieve sustained virological response (SVR). Although the risk of liver tumors is greatly reduced, there are still patients who achieve SVR but will progress to hepatocellular carcinoma (HCC). HCV infection is also a known risk for cholangiocellular carcinoma (CLC), although it is considered a relative infrequent liver malignancy. We report a series of five cases of CLC in patients that achieved SVR after HCV treatment with DAA. There were three women and two males with a median age of 62 years (range 49 to 77 years). Four patients had liver cirrhosis at the time of their HCV treatment. The interval from achieving SVR until CLC diagnosis varied, ranging from 4 to 36 months (median=12). Three patients presented with advanced disease and had extrahepatic spread at the time of their diagnosis. One patient had a resectable tumor, with no recurrence 4 years later. In one case, the tumor was initially considered an atypical HCC and was treated by radiofrequency ablation. Three years later, she was diagnosed with a large tumor recurrence that was demonstrated to be a CLC on liver biopsy. The last two patients were older males with HCV compensated cirrhosis diagnosed with CLC more than two years after achieving SVR. Palliative chemotherapy was started in both. Only a handful of CLC cases have been reported in HCV patients after SVR. Clinicians should take into account the possible development of an aggressive CLC.

## INTRODUCTION

Infection with hepatitis C virus (HCV) is a significant risk factor for liver cirrhosis and hepatocellular carcinoma (HCC) development. The objective of antiviral therapy in HCV infection is the persistent elimination of HCV RNA, known as sustained virologic response (SVR). SVR described as "undetectable" RNA at 12 weeks after the end of antiviral treatment, has been demonstrated to reduce rates of liver decompensation and lower the risk of liver malignancy in patients infected with HCV [1].

Until a few years ago, interferon and ribavirin were the basis of HCV antiviral treatment. The antiviral effect induced by interferon is thought to be associated with an increased response of the host immune system, but it has limited efficacy and important side effects.

In the last five years, the treatment of HCV infection has greatly advanced with the introduction of new, more efficient agents named direct-acting antivirals (DAAs) [2]. These drugs target several nonstructural proteins of the virus that disrupt replication and stop the infection. Compared to interferon treatment, these new drugs are well tolerated with few, if any, side effects and can also be given in patients with decompensated cirrhosis.

Some patients who achieved SVR after DAA treatment still develop HCC. Cholangiocellular carcinoma (CLC), on the other hand, is considered a relative infrequent liver malignancy.

Although several papers have concentrated on HCC development after achieving SVR, to our knowledge, there have been only three reports published concerning intrahepatic CLC associated with SVR after DAA treatment [3–5]. We report a series of five cases diagnosed with intrahepatic CLC who had previously achieved SVR with DAAs.

## Material and Methods

All patients diagnosed with CLC after SVR and treated at our hospital, a tertiary referral center, between 2016 and 2021, were included in this analysis. Demographics (age, gender), initial presentation, location and spread of the tumors, tumor characteristics, treatment and outcome variables were collected.

## Results

Between 2016 and 2021, five patients were diagnosed with CLC after DAA treatment at our institution. There were three women and two males with a median age of 62 years (range 49 to 77 years). Four patients already had liver cirrhosis at the time of their HCV treatment. Two patients had no symptoms and were diagnosed during routine post-treatment follow-up. In two patients, the primary symptom was right upper quadrant pain, and one patient developed jaundice and pruritus. Three patients presented with advanced disease and had extrahepatic spread at the time of their diagnosis.

**Figure 1:**
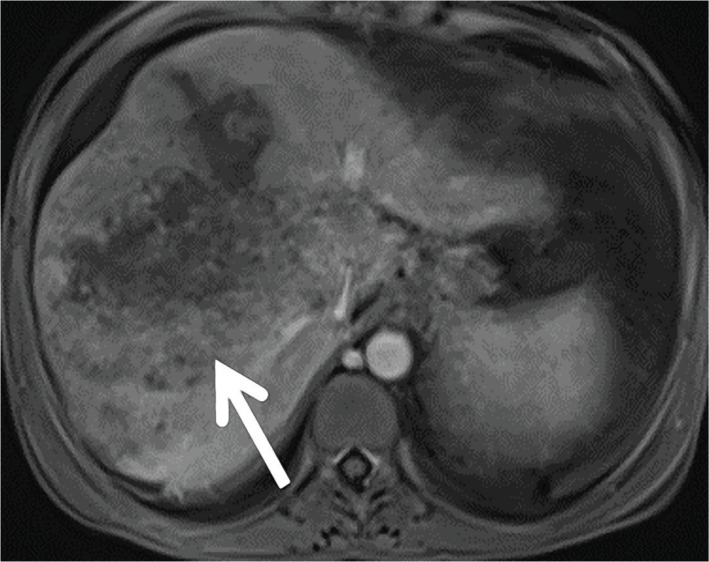
Abdomen MRI revealing a large lesion 8.4×9 cm heterogeneous right hepatic mass with central necrosis and satellite nodules.

**Figure 2:**
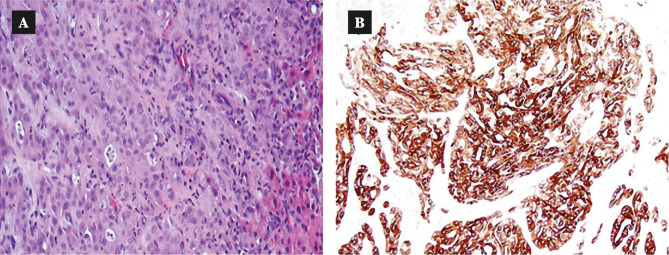
A – Hematoxylin and eosin staining of liver biopsy, demonstrating moderately differentiated adenocarcinoma with very poorly organized glandular structures. B – Immunohistochemistry of liver biopsy, demonstrating positive CK7 staining.

The first patient is a 45-year-old woman with compensated HCV cirrhosis who was treated for 6 months in 2016 with the combination of ombitasvir/paritaprevir/ritonavir and dasabuvir plus ribavirin, achieving SVR. Five months later, an abdominal MRI performed for HCC screening demonstrated a large, 8.5 cm heterogenous, partial necrotic liver tumor, satellite nodules and multiple abdominal adenopathies (Figure 1). Alpha-fetoprotein (AFP), carbohydrate antigen 19-9 (CA19-9) and carcinoembryonic antigen (CEA) were negative. A liver biopsy was performed and showed poorly differentiated adenocarcinoma. Immunohistochemistry staining was positive for cytokeratin (CK) 7, but otherwise, negative (Figure 2). These findings were supportive of intrahepatic CLC. The patient developed peritoneal carcinomatosis and ascites and was enrolled in hospice care. The patient died three months later.

**Figure 3:**
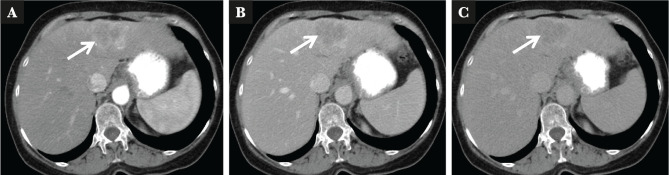
CT abdomen revealing a 3 cm lesion in the left liver lobe A – arterial phase; B – venous phase; C – portal phase.

**Figure 4:**
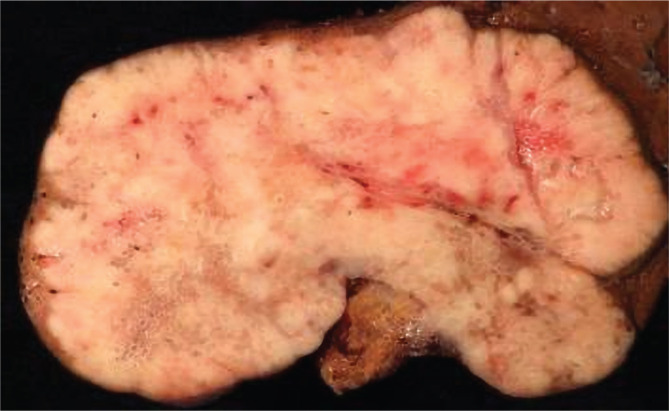
Macroscopically, a single whitish nodular lesion, measuring 4×3.5 cm, with regular margin and capsule.

**Figure 5:**
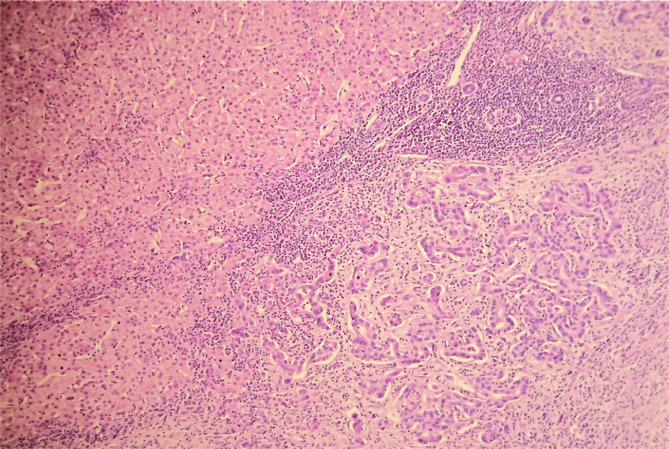
Hematoxylin and eosin staining, tumor cells, with the formation of small and irregularly tortuous glands in the tumor tissues. The noncancerous hepatic tissue was classified as having minimal activity with moderate fibrosis (F3).

A 62-year-old woman was admitted to our department with a 3 cm tumor in the left liver lobe, which had been detected by ultrasound for HCC surveillance. She was diagnosed with compensated HCV cirrhosis and treated with sofosbuvir/ledipasvir 6 months with SVR one year earlier. On admission to our hospital, she had no symptoms, with a physical examination showing no abnormal findings. Blood tests were normal except for mild hypercholesterolemia. Serologic analyses were negative for hepatitis B virus (HBV) and showed reactivity for HCV antibody with undetectable HCV RNA. Tumor markers were negative for AFP (2.2 ng/mL) and prothrombin induced by vitamin K absence (PIVKA-II, 23 mAU/mL), but there was an increased value of CA19-9 (330.7 U/mL). A CT scan revealed a single 3 cm tumor in the left liver lobe (Figure 3). An open left lateral sectionectomy of the liver was performed. Macroscopically, there was a single whitish encapsulated tumor, measuring 4×3.5 cm, with regular margins (Figure 4). Histologic examination revealed malignant cells that form small glands in the tumor tissue (Figure 5). Immunohistochemically, tumor cells stained positive for CK7 and CK19 (image unavailable). Considering these findings, a diagnosis of well-differentiated CLC was established. The noncancerous liver tissue was demonstrated to have minimal activity with moderate fibrosis (F3). The patient is alive with no recurrence five years later.

The next patient is a 59-year-old woman with an HCV infection since 2014 who was treated with pegylated interferon plus ribavirin without SVR. She was negative for prior HBV infection, denied alcohol abuse and did not smoke. Physical examination showed no specific findings. Prior to initiating DAA treatment, a transient elastography examination (Fibroscan) revealed F2 fibrosis.

**Figure 6:**
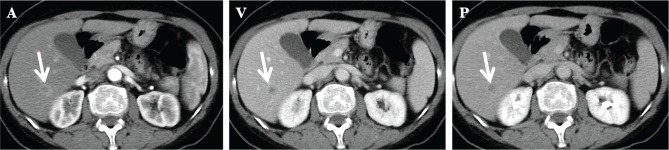
CT abdomen showing an ill-defined low-density area in sg VI with hyper-enhancement in the early phase and iso-enhancement in the late phase A – arterial phase; V – venous phase; P – Portal phase.

**Figure 7:**
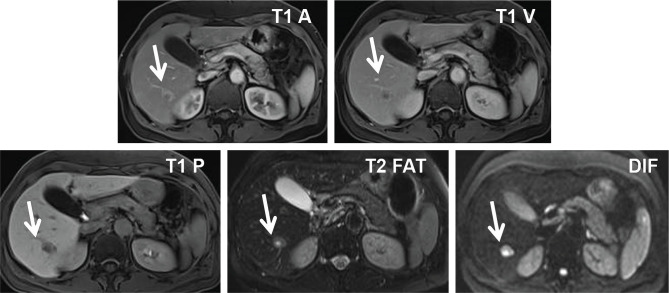
MRI scan, nodule in segment VI showing low intensity on T1-weighted images, high intensity on T2-weighted images, and high intensity in diffusion-weighted images. Images from left to right: T1 arterial, T1 venous, T1 portal, Fat saturated T2, Diffusion.

She was started on ombitasvir/paritaprevir/ritonavir and dasabuvir plus ribavirin for 3 months in 2016 and achieved SVR. Routine ultrasonography performed 6 months later revealed a 17 mm hypoechoic lesion in segment VI. A computerized tomography (CT) demonstrated a small tumor in sg VI with arterial phase hyper-enhancement and later phase iso-enhancement (Figure 6). On magnetic resonance imaging (MRI), the tumor had low signal intensity on T1 images, in combination with high intensity on T2 and on diffusion-weighted images. A dynamic enhanced Gadolinium-ethoxybenzyl-diethylenetriamine pentaacetic acid (Gd-EOB-DTPA) MRI demonstrated arterial hypervascularity and hypointensity in the hepatobiliary phase (Figure 7). Considering all the clinical and imaging findings, a diagnosis of atypical HCC was established. The patient refused a liver biopsy and was treated by radiofrequency ablation. The patient followed hepatology appointments from 2017 until 2019 and underwent repeated liver imaging with no tumor recurrence.

**Figure 8:**
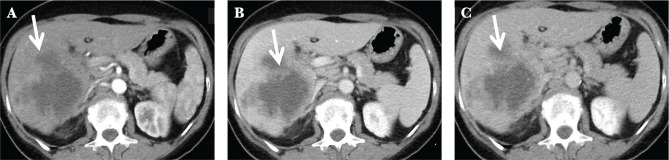
CT abdomen showing a heterogeneous hypodense 5 cm in size mass in the liver, several satellite lesions and abdominal adenopathies A – arterial phase; B – venous phase; C – portal phase.

**Figure 9:**
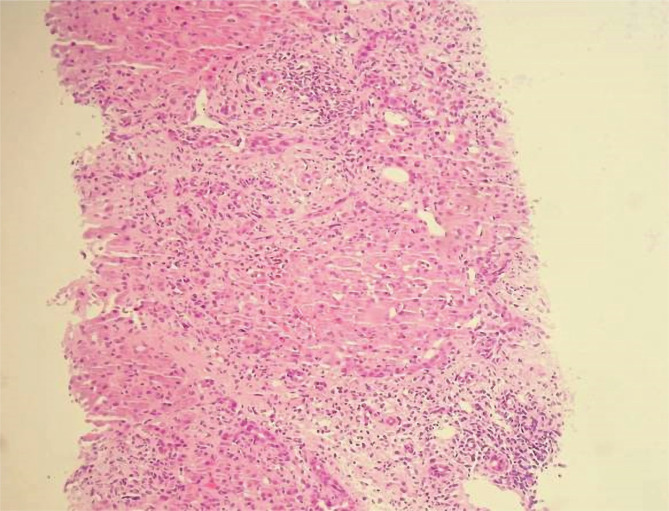
Hematoxylin and eosin staining of liver biopsy, showing moderately differentiated adenocarcinoma with poorly organized glandular structures. Peritumoral tissue with moderate fibrosis, inflammation and ductular reaction.

She missed all appointments in 2020 due to the COVID-19 pandemic and only came in January 2021 when she was diagnosed with a 5 cm liver tumor, several satellite lesions and abdominal adenopathies (Figure 8). A percutaneous liver biopsy was performed with an aspect of CLC (Figure 9), and chemotherapy treatment was started.

The fourth patient is a 65-year-old male treated for HCV compensated cirrhosis with DAA in 2017 with SVR. Three years later, he was admitted for jaundice. On clinical examination, he was jaundiced and had severe pruritus without abdominal pain. Prior medical history consisted of benign prostatic hyperplasia and arterial hypertension; both controlled with therapy. Family history was significant, with two relatives with cancer, the father with colon cancer and the maternal grandmother with gastric cancer.

The patient's liver function tests showed abnormal results, with increased total bilirubin of 11.9 mg/dL, alkaline phosphatase of 1328 IU/L, and elevated transaminase levels. The CA19-9 was mildly elevated to 63 U/mL. The endoscopy and colonoscopy were negative. He was negative for hepatitis B and had hepatitis C antibodies with undetectable HCV RNA.

**Figure 10:**
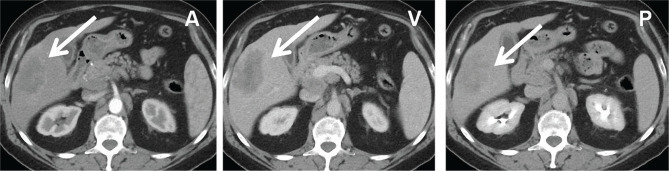
CT demonstrating large liver tumor, hilar adenopathies that cause biliary obstruction and multiple metastatic spinal lesions A – arterial phase; V – venous phase; P – Portal phase.

**Figure 11:**
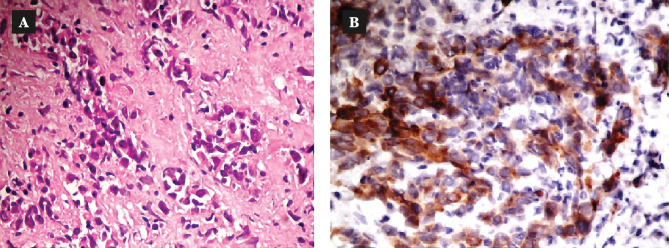
A – Hematoxylin and eosin staining of liver biopsy, demonstrating anaplastic adenocarcinoma; B – Immunohistochemistry of liver biopsy, demonstrating positive for CK MNF 116.

Computed tomography (CT) scan revealed a large liver tumor, hilar adenopathies that cause biliary obstruction and multiple metastatic spinal lesions (Figure 10). Percutaneous biliary drainage was performed with successful internalization with endoscopic retrograde cholangiopancreatography (ERCP). A biopsy of the liver lesion established the diagnosis of undifferentiated CLC. Tumor cells were negative for CK-7, CK-20 and hepatocyte antigen and positive only for CK MNF 116 (Figure 11). Palliative treatment with a combination of gemcitabine and cisplatin was started. The patient deteriorated and decided to stop chemotherapy and enroll in hospice care. He died 7 months later.

**Figure 12:**
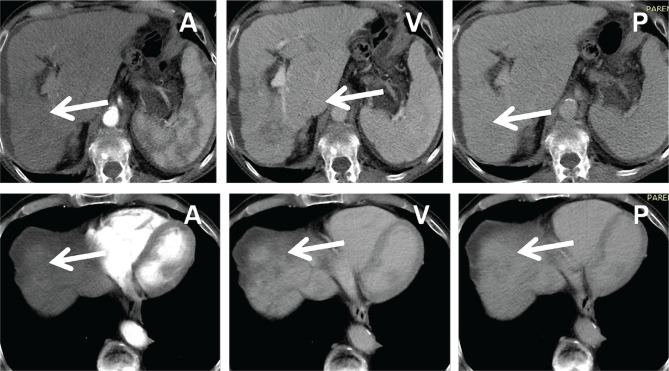
CT scan showing multiple heterogeneous small liver masses A – arterial phase; V – venous phase; P – Portal phase.

The last patient, a 78-year-old male with a prior medical history of coronary artery disease and total gastrectomy for gastric lymphoma and adjuvant chemotherapy, presented for HCV treatment in 2016. He was previously treated with pegylated interferon plus ribavirin but did not achieve SVR. He was negative for hepatitis B surface antigen and positive for HBV core antibody and HBV surface antibody, with undetectable HBV DNA. Before starting the DAA treatment, transient elastography (FibroScan) revealed F4 fibrosis. The patient was treated with sofosbuvir/ledipasvir for 12 weeks, achieving SVR. He was followed up regularly and underwent imaging studies every 6 months. Approximately 2 years after the completion of treatment, he was evaluated for HCC surveillance and discovered to have an elevated CA 19-9 (700 UI/mL), negative AFP and liver function tests within normal limits. He had performed a CT scan 6 months prior that showed no liver tumors. He underwent an abdominal and pelvis CT (Figure 12) which showed multiple heterogeneous small liver masses. A CT scan-guided liver mass biopsy was performed and demonstrated adenocarcinoma with immunohistochemical staining positive for CK7 and negative for CDX2 and CK20. Histologic pictures are not available. Diagnosis of intrahepatic CLC was established based on radiology studies, histology, and immunohistochemical staining. The patient started palliative chemotherapy with gemcitabine and cisplatin. Patients' data are summarised in Table 1.

**Table 1:** Clinical data of patients diagnosed with CLC.

	Case 1	Case 2	Case 3	Case 4	Case 5
**Age (yr)/gender**	49/F	62/F	59/F	65/M	77/M
**Treatment for HCV**	3D+ Riba	3D+ Riba	Sofosbuvir/ledipasvir	3D+ Riba	Sofosbuvir/ledipasvir
**Liver disease**	Cirrhosis	Cirrhosis	Hepatitis	Cirrhosis	Cirrhosis
**Symptoms and signs**	RUQ pain Hepatomegaly	None	RUQ pain Hepatomegaly	Jaundice Pruritus Hepatomegaly	None
**Time after SVR (months)**	6	12	4	36	24
**Liver lesions**	Multiple	1	1	1	Multiple
**Metastases**	Peritoneal	None	None	Bone	None
**Treatment after diagnosis**	Hospice care	Surgery	RFA Gemcitabine/cisplatin	Gemcitabine/cisplatin	Gemcitabine/cisplatin
**Survival**	Dead after 3 months	Alive 4 years	Alive 12 months	Dead after 7 months	Alive 6 months

## Discussion

Cholangiocellular carcinoma (CHC) is the second most frequent primary malignant liver tumor after hepatocellular carcinoma (HCC), with a variable incidence between 5% and 25%. It can be classified into two groups, intrahepatic CLC and extrahepatic CLC [6].

Cholangiocellular carcinoma was initially characterized by Steiner and Higginson in 1959. Its origin was considered to be the canals of Hering, which are located between the interlobular ducts and bile canaliculus [7].

Globally, intrahepatic CLC is known to have a wide incidence variability and geographically distinct risk factors. Europe and North America have historically been considered low incidence areas for CLC, but the number of cases has steadily risen in the last decades [8].

Besides widely recognized risk factors (primary sclerosing cholangitis, hepatolithiasis and liver fluke infestation) that promote CLC development, different studies have assessed the relationship between viral hepatitis infections and this type of tumor.

HBV and HCV infections and heavy alcohol consumption are known risk factors for HCC, which is a fast-rising cause of cancer-related death worldwide [9]. While their roles as risk factors in CLC development are not clearly established, in recent years, several studies have linked HCV infection and liver cirrhosis with the development of CLC [10, 11].

In a Japanese prospective cohort study in patients with cirrhosis due to HCV infection, 2.3% developed intrahepatic CLC during a mean follow-up period of 7.2 years. The same authors demonstrated that patients with HCV cirrhosis had a 1,000 times higher risk of developing intrahepatic CLC than the general population [12]. A meta-analysis of HCV patients who developed intrahepatic CLC demonstrated a significant association between HCV infection and the incidence of intrahepatic CLC [13]. According to one study, in infected patients with either HBV or HCV, the incidence rate of HCC to intrahepatic CLC is 13.7 to 1 [14]. HCV infection associated with intrahepatic CLC may still be underrated compared to the association with HCC [13]. On the contrary, there are other reports in which more than half of intrahepatic CLC patients had no risk factors associated with chronic liver disease [15, 16].

Because the exact mechanism of CLC development remains largely unclear, different carcinogenic processes in patients with chronic HCV infection have been suggested. It has been demonstrated that the hepatitis C virus can cause injury to cholangiocytes, leading to a range of inflammatory, proliferative, and degenerative damage. Similar to other pathological conditions that increase the risk for CLC development, long-standing chronic inflammation and regenerative hyperplasia of the bile duct epithelium can induce malignant transformation.

Until recent years, HCV infection treatment associated with pegylated interferon plus ribavirin has been challenging, with significant side effects for patients and low rates of SVR.

A finer understanding of the viral replication mechanism has led to the developing of new treatment agents called direct-acting antivirals (DAAs) that are effective against nonstructural viral proteins. DAAs are now extensively used in clinical practice to treat patients with chronic HCV infection in all stages, from hepatitis to decompensated cirrhosis, with very high SVR rates [17].

The safety of DAA treatments has been demonstrated in clinical trials, with only minor side effects like headache or nausea being reported. Considering their efficacy and tolerability, these drugs have been widely adopted, although long-term outcomes or possible complications are not fully known.

After introducing DAAs in clinical practice, some initial reports showed an apparent increase in *de novo* rates of HCC in treated patients with SVR. Some authors also linked DAA treatment to an early tumor recurrence after loco-regional therapy in HCC patients [18]. Multiple, mostly retrospective case series comparing HCC recurrence rates to older records in patients with pegylated interferon therapy and also meta-analyses investigating these issues were published. Currently, larger multicenter studies have demonstrated no increased risk of HCC development after DAA therapy [19, 20]. Earlier reports may be explained by the fact that more patients with advanced chronic HCV disease received antiviral than in the past, which increases the general risk for HCC development without any relation to a specific treatment regimen. Until now we have found only three published case reports regarding the occurrence of intrahepatic CLC after DAA treatment [3–5].

We consider that our report is especially significant because none of our patients was known with prior liver malignancy, which demonstrates that these are *de novo* tumors.

Interestingly, all five cases were intrahepatic CLC, which comprises only 8% of total cholangiocarcinomas [21]. In three cases, the tumors were identified on examinations performed for HCC surveillance. The time interval between the end of the antiviral treatment and the detection of malignancy extended from four to 36 months. In our series, two patients already had distant metastasis when the intrahepatic CLC diagnosis was established, which suggests a very aggressive course.

We consider that five cases of intrahepatic CLC among HCV-treated patients in one center in two years are significant since three cases were diagnosed more than a year after successful DAA treatment.

On ultrasonography, intrahepatic CLC usually appears as a heterogeneous hypoechoic mass with an imprecise border and without bile duct dilatation. On MRI, CLC usually shows low intensity on T1 images and high intensity on T2 and diffusion-weighted images, but in some cases, it can also have hyper-enhancement of the periphery or the entire lesion in the early phase and hyper-iso-enhancement in the delayed phase. Additionally, intrahepatic CLC, in most cases, demonstrates a defect in the hepatobiliary phase on Gd-EOB-DTPA-enhanced MRI [22]. In our paper, four patients had imaging findings similar to previously described features of intrahepatic CLC. In patient number three the initial imaging diagnosis was not correct. The tumor initially diagnosed as an atypical HCC was, in fact, histologically, a small intrahepatic CLC.

Slowly growing CLC, over several years, have been described in a small number of case reports [23]. In the first two patients, we cannot clearly determine that the intrahepatic CLC had already existed at the time of SVR achievement. However, ultrasonography did not show any suspicious lesion at least 3 months prior to the discovery of the tumor on MRI. This emphasizes that hepatic tumors should be excluded through constant imaging evaluations, even in younger patients achieving SVR.

Histopathological examination of the first three cases consisted mainly of narrow bile duct structures composed of atypical cells, with immunohistochemistry showing tumor cells positive for CK7 and CK19 and negative for hepatocytes markers. The fourth case was an anaplastic carcinoma considered a poorly differentiated cholangiocarcinoma.

Surgical resection is the recommended treatment method but is contraindicated in cases with multiple tumors or if the extrahepatic spread is present [24]. Only one patient in our series was suitable for resection after the initial diagnosis. In our hospital, the dissection of the hilar lymph nodes is done for preoperative diagnosed CLC in cases where there is a suspicion of metastatic involvement. In our case, lymph node dissection was not done since there was no suspicion of being metastatic on either imaging or intraoperatively. Adjuvant chemotherapy could be considered in the future regarding the possibility of regional lymph node recurrence. The patient is currently examined by MRI and tumor markers every 6 months. Because there are no signs of recurrence, for the moment, we do not take into account any additional treatments.

Intrahepatic CLC has a poor prognosis in HCV carriers, as previously demonstrated [25]. In our series, all patients except one had a very poor outcome, with a survival of less than a year.

Currently, treating the infection is the simple part of HCV care, as DAA treatment is safe and can cure almost all patients in 8 to 12 weeks. Treating the complications of cirrhosis and especially liver tumors over time can be more problematic. Although there is still no clear explanation for tumor development in patients with SVR after DAA treatment, one theory states that an abrupt reduction of the viral load can cause a reduced intrahepatic immune activation and diminish tumor surveillance [26]. Taking this into account, it can be assumed that an abrupt elimination of HCV can cause the disappearance of the apoptotic effect induced by viral proteins in the host cells. This fact can lead to the unrestricted proliferation of damaged hepatic cells.

An *in vitro* study that investigated the effect of sofosbuvir and daclatasvir on HCC and CLC-derived cell lines suggested the possible occurrence of off-target effects that can modulate cell proliferation, invasion capability and gene expression [27]. Off-target effects can induce phenotypic changes associated with concomitant variations of gene expression that induce the reduction of malignancy-risk demonstrated in most patients but might also explain some rare instances of particularly aggressive tumors after DAA treatment.

As a recent consensus paper clearly demonstrated that liver cirrhosis and HCV infection are both important risk factors for intrahepatic CLC [8], we think that our cases might be explained by the advanced stage of the liver disease at the time of the antiviral treatment rather than an effect of the DAA treatment itself.

To summarise, we are far from a complete understanding of all the effects of DAA treatment in patients with advanced liver disease and we think this should be investigated in the future. We encourage other clinicians that treat patients with hepatic malignancies after HCV eradication to report similar experiences. We consider that tumor surveillance in all cirrhotic patients who achieve SVR after DAA treatment should be continued even in cases with fibrosis regression. Research on the link between HCV cirrhosis treated with DAA and CLC still remains ill-defined and deserves attention.

## Conclusion

Intrahepatic cholangiocellular carcinoma seems to be an aggressive malignancy associated with HCV infection. Cirrhotic patients can still develop cholangiocellular carcinoma years after achieving SVR, even in cases with fibrosis regression. Further research on the impact of SVR on all HCV-associated malignancies is needed.
